# ‘Working is out of the question’: a qualitative text analysis of medical certificates of disability

**DOI:** 10.1186/s12875-017-0627-z

**Published:** 2017-04-20

**Authors:** Guri Aarseth, Bård Natvig, Eivind Engebretsen, Anne Kveim Lie

**Affiliations:** 1 Department of General Practice, University of Oslo, Faculty of Medicine, Institute of Health and Society, Postboks 1130, Blindern, 0318 OSLO Norway; 2 Department of Health Science, University of Oslo, Faculty of Medicine, Institute of Health and Society, Postboks 1089, Blindern, 0318 OSLO Norway; 3 Departement of Community Medicine and Global Health, University of Oslo, Faculty of Medicine; Institute of Health and Society, Postboks 1130, Blindern, 0318 OSLO Norway

**Keywords:** Norway, GP, Medical certificates of incapacity for work, Document analysis

## Abstract

**Background:**

Medical certificates influence the distribution of economic benefits in welfare states; however, the qualitative aspects of these texts remain largely unexplored. The present study is the first systematic investigation done of these texts. Our aim was to investigate how GPs select and mediate information about their patients’ health and how they support their conclusions about illness, functioning and fitness for work in medical certificates.

**Methods:**

We performed a textual analysis of thirty-three medical certificates produced by general practitioners (GP) in Norway at the request of the Norwegian Labour and Welfare Administration (NAV).The certificates were subjected to critical reading using the combined analytic methods of narratology and linguistics.

**Results:**

Some of the medical information was unclear, ambiguous, and possibly misleading. Evaluations of functioning related to illness were scarce or absent, regardless of diagnosis, and, hence, the basis of working incapacity was unclear. Voices in the text frequently conflated, obscuring the source of speaker. In some documents, the expert’s subtle use of language implied doubts about the claimant’s credibility, but explicit advocacy also occurred. GPs show little insight into their patients’ working lives, but rather than express uncertainty and incompetence, they may resort to making too absolute and too general statements about patients’ working capacity, and fail to report thorough assessments.

**Conclusions:**

A number of the texts in our material may not function as sufficient or reliable sources for making decisions regarding social benefits. Certificates as these may be deficient for several reasons, and textual incompetence may be one of them. Physicians in Norway receive no systematic training in professional writing. High-quality medical certificates, we believe, might be economical in the long term: it might increase the efficiency with which NAV processes cases and save costs by eliminating the need for unnecessary and expensive specialist reports. Moreover, correct and coherent medical certificates can strengthen legal protection for claimants. Eventually, reducing advocacy in these documents may contribute to a fairer evaluation of whether claimants are eligible for disability benefits or not. Therefore, we believe that professional writing skills should be validated as an important part of medical practice and should be integrated in medical schools and in further education as a discipline in its own right, preferably involving humanities professors.

## Background

Physicians are producers of texts, although they are probably not inclined to consider themselves as such. Written reports go hand in hand with clinical work, as they have since the Hippocratic writers communicated their clinical observations in case reports. In Europe, the medical case report was refined and ‘purified’ during the 18th and 19th centuries, becoming a professional skill in its own right [1, 2]. In our time, society relies on medical texts to approve, testify to, and document nearly everything that can be related to ‘health’. Accordingly, the amount of administrative documentary work that doctors must perform is increasing at the expense of their clinical work [3]. In doing this work, physicians are supposed to exit the mode of treating doctor and take on the role of expert, balancing the perspectives of professional sympathy and impartiality [4].

A certificate – from the Latin *certificare*, meaning to guarantee as certain or attest in an authoritative manner (Oxford English Dictionary) – verifies facts; a medical certificate, in particular, attests to the condition of a person’s health. It can be used to legitimise benefits to compensate for loss of income as the result of disease, or to exempt or relieve a person from his/her normal activities. In this article we examine medical certificates written by Norwegian GPs, taking disability benefits as our point of departure. Our aim is to investigate how these physicians, when acting as experts, mediate their patients’ illnesses, functioning, and fitness for work and how they report assessments made on a medical basis. Our purpose is to encourage a discussion about the role of GPs in the disability benefit scheme and address the need for a systematic incorporation of writing instruction in medical schools and in further education.

Medical certificates play a crucial role in allocating benefits in welfare states. To be legal, a medical certificate must generally be issued by authorised health personnel. The authority of the document depends on an authorised reader sharing the perception of its factual veracity [5]. A certifier must provide verifiable information; the certificate must be accurate, not misleading or flawed, and should be written based on the physician’s recent, personal examination of the patient.

In Norway, according to the National Insurance Act, a medical certificate of disability must make probable the causal connection between a patient’s illness, their loss of function, and their need for permanent income compensation [6]. In issuing these certificates, therefore, GPs have to navigate a problematic landscape in which persistent complaints, symptoms, and illness, with or without objective correlatives, must be subjected to the certifier’s professional reasoning. GPs recognise that they have little knowledge about patients’ workplaces, their functioning, or their working capacity [7–12]. In addition, formal medical knowledge may be insufficient as a basis for determining individuals’ rights and eligibility for social benefits [7].

Arthur Kleinman [13] defines *illness* as the ‘innately human experience of symptoms and suffering’, *disease* as the practitioner’s ‘recasting of illness in terms of theories of disorders’, and *sickness* as the understanding of a disorder (for instance tuberculosis) across a population in relation to macrosocial forces (poverty, education etc.). In contrast to this English triad, the Norwegian term *sykdom* comprises both illness and disease (in the National Insurance Act, *sykdom* also refers to injury [*skade*], impairments, and defects [*lyte*]) [14].

The ethics of issuing medical certificates differ in several respects from those of producing clinical documents. First, the information contained in a certificate is, in part, exempt from obligations to protect patient confidentiality. Second, whereas as therapists physicians are expected to always consider the best possible outcome for their patients, as experts they should act according to criteria of objectivity. In other words, physicians should provide information that is impartial, correct, verifiable, and accurate, [4], and act ‘without regard to the outcome of the case’ [15]. Moreover, physicians should not be passive mediators of the patient’s concern and utterances, but act as a ‘performer of the profession’ [4].

The ethical and professional dilemmas of physicians facing the discourses of both law (including the gatekeeper function of the doctor) and medicine are treated in studies of GPs’ practices of certifying sick leave [16–19]. In a questionnaire survey, Gulbrandsen et al. [20] found that it is not uncommon for Norwegian GPs to provide medical certificates strategically written in favour of the patient; however, the GPs in that study claimed that they were not twisting the facts or lying, but rather using discretion and argumentation. Other investigations have indicated that GPs may be directed by advocacy and the patient’s subjective needs and wishes when issuing sickness certifications [17, 21, 22], as well as by their own personal values and attitudes [23], personality, and beliefs [24].

Direct investigation of the content of certificates is scarce, but Kiessling et al. [25] found that 90 per cent of a selection of Swedish medical certificates lacked relevant or adequate information about the patient’s ability to function and capacity to work; others have shown that ambiguous statements about a patient’s medical disorder are not uncommon [26]. In a previous article we presented a linguistic analysis of the same medical certificates of fitness to work which showed that certifying GPs tended to emphasise the patient or claimant principally as a passive carrier of symptoms with little or no agency [27]. The above studies indicate that ambiguities, possible biases, and a lack of relevant information may undermine the validity of decisions made on the basis of medical certificates.

In addition, officials processing disability benefits cases within the Norwegian Labour and Welfare Administration (NAV) experience that deficient or unclear information in GPs’ medical certificates can lead to costly and time-consuming delays [28]. In such cases, additional information needs to be obtained; this information is increasingly (though probably unnecessarily) ordered from specialists charging significantly higher fees. Because the specialist needs to see the claimant personally and write a new note, it usually leads to an increase in the case’s processing time, sometimes by several months. The delay creates temporary uncertainty for the claimant as to the outcome of the proceeding.

Compared to GPs in some other European countries, GPs in Norway play a prominent role in assessing patients’ health-related inability to work [29] and, thus, are highly influential in the distribution of public resources. Their importance can be illustrated by the fact that presently 9.5 per cent (317 700) of the population between 18 and 66 years of age [30] are living on disability benefits. Altogether, the largest group of benefit recipients – 63 per cent – have been diagnosed either with diseases related to the musculoskeletal system (mainly low back pain and fibromyalgia) or with mental disorders (mainly anxiety and depression) [31, 32]. The government estimate of the total costs of working disability benefits in Norway in 2016 was 78.2 billion NOK (€8.55 billion) [33]. Decisions on disability benefits are taken by non-medical officials in the regional offices within NAV, who never meet the claimant personally and must rely entirely on textual information about that person.

In this article, using narratological and linguistic analysis, we examine a sample of texts written by GPs in their role as experts to determine how they communicate the illness, functional ability, and working capacity of people claiming disability benefits.

## Methods

### Data collection

Our material consists of medical certificates of incapacity for work produced by Norwegian GPs. They are, with one exception (which was written by freehand), written on a standardised form, the Medical Certificate of Disability [34]. The certificates were collected in the period March–June 2013; all of them were written between 2007 and 2013. At the time of collection, the cases had already been assessed and closed by NAV. NAV’s central statistical register of assisted us in selecting 150 potential certificates within four counties in Norway.

We strategically selected individuals based on age, sex, geographic region, and diagnosis. The age range was 18–66 and at least one third belonging to one sex. One third of the certificates fell into the category of musculo-skeletal related diagnoses, one third into the category of psychiatric diagnoses, and the remaining third into the category of other disorders; thus the sample roughly reflects the distribution of diagnoses among the total population of Norwegians receiving disability benefits. The four counties were selected according to the proportion of the population receiving disability benefits: one had a relatively high proportion (>12%), two were in the median (8–12%) and one had a low proportion (<8%).

The project was approved by the Data Protection Official for Research [35], the Directorate of Labour and Welfare, and the Council of Secrecy and Research in the Ministry of Justice and Public Security. An informed consent letter, also approved by the Data Protection Official for Research, was sent to the 150 claimants via their local NAV offices. We anticipated a final number of 30–50 certificates: forty recipients of disability benefits consented to us accessing their medical certificates. Of the forty certificates, seven were excluded as they were either not about disability or not written by GPs. All the documents were anonymised with respect to the claimants. The number of different certifiers (GPs) equalled the number of certificates. The final sample is shown in Table 1. The main and additional diagnoses complied with the ICPC-2 system (International Classification of Primary Care, Second Edition).

**Table 1:** Characteristics of the medical certificates of disability (*N* = 33)

Geographic region	Regional distribution	
North	12	
Northwest coast	3	
Central South	4	
Central East	14	
Male claimants	20	
Female claimants	13	
Age range of claimants	30–64 (average = median = 45)	
Diagnosis (ICPC-2)	Main:	Additional:
Musculoskeletal	20	17
Psychiatry	6	4
Others:	9	10

### Analysis

We apply qualitative document analysis to our empirical data, which consist of textual units formed by the GPs texts and the form text, Medical certificate for work incapacity (Table 2). Our analysis of the medical certificates draws on the discourses of narratology and formal linguistics. As subjects of analysis, documents are not ‘surrogates’ for reality, but are themselves social facts, exchanged as part of a social interaction [36]. Because documents do not speak for themselves, they must be ‘made to speak by the analyst’ [36]. Hence, what we are investigating is not the patient but the documentary *depiction* of the patient, his/her illness and ability to work. Narrative analysis (as a method) investigates structures and techniques of telling, the narrator’s perspective (who speaks?), and the consistency of the facts and interpretations the narrator provides. We use linguistic methods of analysis at the word and sentence levels to investigate different types of grammatical connections between sentences and their meaning.

**Table 2:** Medical certificate for work incapacity

National Insurance (NAV)	
Medical certificate for work incapacity	
The physician is to send this to the local NAV office.	
0	This certificate concerns:
0.1	Assessment of work capacity at sick leave
0.2	Rehabilitation money
0.3	Disability pension
1.0	Information about the patient and employment
	Name:
	Year of birth:
	Certificate written: date
	Employer’s name and address:
2	Information of diagnosis and disease
2.1	Main diagnosis
2.1.1	Code of diagnosis
2.2	Additional diagnosis
2.2.1	Code of diagnosis
2.3	Classification: ICPC-2/ICD-10
2.4	Completely incapacitated since
2.5	Story of disease, symptoms and treatment
2.6	Current clinical status (specify date). The results of relevant investigations
2.7	Should NAV consider this to be:
2.7.1.	Occupational disease? (Yes/No)
2.7.2.	If yes: date of injury
3	Plan for medical examination and treatment
3.1	Is the patient referred for
	Medical assessment (specify)?
	Medical treatment (specify)?
3.1.1	Date of referral for medical assessment. 3.1.2 Expected waiting time (weeks)
3.1.3	Date of referral for medical treatment. 3.1.4 Expected waiting time (weeks)
3.2	Plan for medical examination. Specify the planned examination and time duration.
3.3	Plan for medical treatment.Specify the planned treatments and time/duration
3.4	Re-evaluation of previous plan of examination and treatment
3.5	When should the NAV office request new medical information regarding work clarification and treatment programme?
3.6	If further treatment is not relevant, give justification
4	Proposed measures beyond medical treatment
	Are the following measures applicable, on a medical basis. Yes/No
	If *yes*, which ones? a) reference to specialist, b) transport subsidy, c) graded sick leave, d) technical aids, f) unemployment benefit, g) others – which ones? Give supplementary information.
	Are there any specific considerations to be made as to these measures?
	If *no*, give justifications
5	Medically reasoned assessment of work ability
5.1	Describe how the patient's functionality is generally reduced because of disease.
5.2	Is the patient engaged in paid work or domestic work, a student, other?
	Specify:
	Briefly describe the type of work and the requirements:
5.3	Assessment of working capacity
	Will the patient be able to
	a) Resume the earlier work (No/Yes)? If yes: now/after treatment
	b) Take other work
5.4	a) What is it that the patient cannot do in the present work?
	b) What other possible considerations need to be taken regarding the choice of another profession/work?
6	Prognosis
	a) Is the treatment assumed to produce an improved ability to work? Yes/No
	b) Estimate the duration of the illness/injury.
	c) Estimate the duration of the functional disability.
	d) Estimate the duration of the reduced working capacity.
7	Causation
	Estimate the importance of the functional disability for the reduced working capacity.
8	Optional information
9	Co-operation/Contact
	Select those that should be contacted by the NAV office: The doctor/employer/NAV/others
10	Reservations
10.1	Is there anything in the certificate that the patient, for medical reasons, should not know? If yes, specify what the patient should not know.
11	The physician’s signature, etc.
11.1	Date, the physician’s name and address
11.2	The physician’s signature
11.3	Telephone number

Narratives, Barbara Smith suggests, can be defined simply as ‘verbal acts consisting of someone telling someone else that something happened’ [37]. The ‘something happened’, which Labov and Waletsky call the ‘complication’ of the story [38] and van Dijk calls the ‘worth telling’ [39], is a key concept of narrative. The ‘complication’ or the ‘worth telling’ is an essential event of the story that has an impact or changes stable situations, conditions or life trajectories. Van Dijk, for instance, emphasises events (disease, in this case) that break the established norms (e.g. of being independent and self-supported) and routines (e.g. daily doings), threaten the basic values (e.g. being healthy) and disturb the balance (e.g. abilities and mastering), plans and goals (e.g. relations, work, education) of the story’s protagonist (who may be an individual with a disease).

Events may be related from different perspectives. Smith points to the inherent relativism of narrative: every narrative is a *version* of some reality, always ‘constructed in accord with some set of purposes or interests’ [37]. Nevertheless, as Petter Aaslestad points out, the ideal starting point of the documentary text is ‘the true event’, as perceived by the narrator [40]. Leaning on the narratological theories of Wayne Booth and Gérard Genette, we distinguish between the ‘historic author’ (the GP), the ‘narrator/writer’ (the telling voice in the text) and the ‘implicit author’ (the text’s norm carrier) [41, 42]. The narrator may represent different perspectives: they may be present as the first person narrator (the ‘I’ of the text) who has limited knowledge of the patient, or as an all-knowing, invisible or impersonal speaker. The intentions of the historic author (the GP) may deviate from those of the narrator for several reasons [43]. Therefore, we distinguish between the GP as a treating family physician on the one hand, and as a narrator on the other. This distinction is also considered in Norwegian legal regulations [44].

The author has in mind an addressee – not a particular, identified person, but rather the author’s idea of a reader, or what Wolfgang Iser terms the ‘implied reader’ [45]. The implied reader is anticipated in the text as a set of the addressee’s competences, reactions, and values [43]. In this case of the medical certificates, the implied reader is represented by (unidentified) NAV employees, including the internal medical adviser. The latter is also an *authorised* reader who shares the knowledge and the terminology of the certifier [43].

The coherence of any given text is associated with consistency, logic, and textual unity. It also depends on the reader’s willingness and ability to create meaningful relations between facts, events, and evaluations [46]. Coherence is thus a product of the text and the reader’s knowledge, experience, attitudes, expectations, and cultural background [46–48]. Some narratologists may include the narrator’s evaluation of facts and events as one of the main elements of a narrative, without which they regard the narrative as pointless [38].

In our analysis, we investigate the narrative thread and the linguistic markers that constitute the global ‘hanging-togetherness’ of the text: the diagnosis, the story of illness, the symptoms belonging to it, and the patient’s ability to function and capacity to work. We use the term illness when talking about the patient’s symptom complex, as claimants of disability benefits are ill or ill-functioning in the sense of having a subjective experience of being unable to work.

## Results

### The standard certificate form: dissociating the disease and the patient

We start with a critical review and interpretation of the form text (Table 2). As a starting point, we ask how the form deals with *disease* and *illness without disease*. On the form, information about the medical condition is requested in terms of ‘diagnosis and information about the disease’ (Section 2); the form breaks this information down into a ‘main diagnosis’ (Subsection 2.1), an ‘additional diagnosis’ (Subsection 2.2), a ‘disease story with symptoms and treatment’ (Subsection 2.5) and ‘results from clinical examination and relevant tests’ (Subsection 2.6). Thus far, the form reflects a model in which *disease* – composed of a set of symptoms, clinical and laboratory findings, and adequate treatment – is the main cause of dysfunction. The form reflects the idea that there is both an immediate and predictable relation between disease and dysfunction/working capacity and a medical treatment available that may improve not the patient’s health but their ‘functional ability’ and ‘working capacity’. This is shown in Subsection 5.1 and Section 6 of the form (see Table 2 for full form):

5.1 Describe how functional ability is generally reduced due to disease.

6. Prognosis:

a) Is the treatment assumed to improve working ability? Yes / No

b) Estimate the duration of the disease/injury

c) Estimate the duration of the functional disability

d) Estimate the duration of the reduced working capacity

Sections 5.1 and 6a–d assume that there is a direct relation between disease and ability to function without the *patient* being viewed as a subject with motivation, beliefs, and a will of their own. Moreover, the form does not ask for the social context (familial, educational) of the claimant, which – according to the National Insurance Act [6] – should be considered a legitimate co-factor of illness. Because an individual’s experience of illness cannot be validated by ‘clinical findings and relevant test results’, important information about the patient and other causes of ill-health may be overlooked. In the standard form, NAV adopts a reductionist view and selects, organises, and limits the information it seeks from the GP, using terms of disease exclusively. This is also seen in Section 2.5, the heading of which – ‘history of disease, symptoms, and treatment’ – indicates that there is a narrative to be told. However, the story being asked for is that of the disease, not of the sick *person.*

### Listing symptoms at the cost of the illness story

Probably as a reflection of the form’s dissociation between the patient and their disease, there are entire certificates that mention the patient only rarely or not at all and which are almost entirely devoid of narratively organised events and their consequences. Excerpt (a), below, exemplifies how the illness story is replaced by a number of symptoms (all the excerpts are from different certificates and are labelled sequentially with letters a-j. The form questions/sections are numbered according to Table 2. Form text is in bold.)

(a) **2.5: History of disease, symptoms and treatment**. Investigated by cardiologist -12 [2012] for a tendency to arrhythmia. Allergies: suggesting wheat flour; gastrointestinal gas. Sensitivity of airways. Afflicted by tinnitus; evaluated [by] the RA [rheumatic arthritis] clinic; Natural functions: a little reflux, ventricular gas, statically: back problems for many years.

Because the list of symptoms/conditions lacks the only element that may connect them – the person to whom they belong – the information comes out fragmentarily, and in the absence of verbs, nothing *happens.* The only traces of a narrative in (a) are ‘-12’ and ‘for many years’, indicating a time course. As Smith points out [37], any utterance, however small and fragmentary, can have narrative content, and ‘telling about events’ can be indistinguishable from stating that ‘something is’. Are the conditions listed – allergies, tinnitus, back problems etc. – events of the past or are they still causing low functioning and working disability? There is nothing in the text that tells us. Listing a large number of symptoms might be the writer’s attempt to comply with NAV’s notion of *disease* as the legitimate basis of disability benefits. What the narrative’s ‘worth telling’ is, however, remains unclear. A mere list without specification or evaluation does little to help up comprehend the patient’s problem, and we are left with a pointless story [38]. The reader whom the text addresses will probably have difficulty interpreting the information and taking a decision; indeed, in this particular case, the disability benefits claim was rejected by the NAV.

### Lack of temporal aspects of illness obscures causality

Because an illness *story* can be abbreviated, told fragmentarily, or not told at all, as shown in excerpt (a), the sequential events are often omitted. In (b), the undefined time aspect of the claimant’s alcoholism and anxiety disguises a possible problematic relation between the patient’s alcohol consumption and their psychiatric disorder.

(b) **2.5 History of disease, symptoms and treatment**. The patient has a long illness story of alcohol abuse. In addition, she has been struggling with anxiety problems for as many years.

The use of ‘in addition’, a simple transitional phrase, indicates that there is no *causal* relation between alcohol abuse and anxiety; however, a competent reader probably cannot help but pose the question: does she have anxiety problems because of alcohol abuse – or is it the other way round? The answer to this question has implications for the treatment of each of the problems. Again, the distinction between what *was* and what *is* is blurred, and the illness textually slides into a continuous, non-contextualised state of being where anxiety and alcoholism are depicted as if they were not related.

### Chronicity and the doctor’s chaos narrative

The medical profession is typically oriented diachronically; taking patient histories is heavily emphasised in the teaching of medical students. Throughout clinical examinations and treatment, the patient’s story – as noted in their medical record – is transformed into medical discourse; but principally it remains a *story* [49]. The medical certificates in our sample, however, are mainly synchronic; it describes the patient’s present symptoms and complaints as a persistent, static state of affairs. According to the sociologist Arthur Frank, disease starts with an event ‘interrupt[ing] life’ and in some cases slides into an ‘incessant presence with no memorable past and no future worth anticipating’ [50].

Medical certificates of disability typically present illness as a *status quo*, which is coherent with the purpose of the document. Furthermore, details of the patient’s condition and story are often omitted. We see an example of this in excerpt (c): it starts in the present, describing the illnesses in very general terms

(c) **2.5 History of disease, symptoms, and treatment**. The patient has a complex health problem with fibromyalgia and extensive muscle pain, […] asthma and general exhaustion.

The terms ‘complex’, ‘extensive’, and ‘general’ seem to replace a thorough depiction of the patient’s illness story which becomes a condition with no pre-history (how was life before the illness started? How and when did the asthma influence her functioning? When – and perhaps why– did she become generally exhausted?).

Arthur Frank [50] has elaborated three empirical categories of illness narratives: first, there is the restitution narrative (the successfully ill – I was healthy, got sick, recovered); second, the quest narrative (the acceptance of illness and how to see the experience as meaningful for oneself and others); and, finally, the chaos narrative (the persistent disturbance to living caused by illness). According to Frank, the chaos narrative – as opposed to the restitution and quest narratives – is distinct in that it has ‘no sequence’ and ‘no mediation, only immediacy’ [50]. In contrast to stories in which medicine triumphs, Frank asserts, the chaos narrative is a non-plot of ‘never getting better’ that exposes the vulnerable, futile, and impotent sides of the profession. In excerpt (d), below, the writer describes a 59-year-old woman with chronic muscle and joint pain who has no authoritative diagnosis to explain the pain, and, consequently, has been denied disability benefits:

(d) **Diagnosis:** Low back pain L03. Pain in joints IKA L20.

**2.5. History of disease, symptoms, and treatment** […] much pain and low function of the shoulder and she was referred to [polyclinic]. There, they found tendinitis. Recommended physiotherapy. The patient has consistent, chronic pain and is treated regularly without improvement of her functioning and there is no curative treatment.

**2.6. Current clinical status (specify date). The results of relevant investigations.** The patient comes for a talk. […] Taking new blood tests and glucoses. Supporting talk. Follow-up of results. Try to find discharge summary from the chiropractor. Additionally, she gets a referral to the physiotherapist.

After the introduction, the narrator enters the text as a first-person narrator (although first- person pronoun is omitted), as a GP acting in present time (probably in a consultation). While the activities described here are normal activities in a family practice – the text is most likely cut and pasted from the patient’s record – they are hardly *worth telling* in a medical certificate. The GP acting in the text, however empathic, does not seem to have a plan: even though therapy has proved to be without effect, the doctor continues to refer the patient to physiotherapy and recommends diverse medical checks not quoted in the excerpt (among them, another MRI). The GP in the text might accept that there is no ‘diagnosis’ and that further treatment and repeated MRIs will not bring more clarity. Instead, this would be a situation that has ‘no mediation, only immediacy’ [50]. An aimless narrative in a certificate may mirror the author’s own ‘chaos’, the GP’s experience of powerlessness when faced with illness without disease.

According to Sarah Nettleton, patients with medically unexplained symptoms (MUS) typically present illness without a clear ‘plot’ or ‘route map’, and, in addition to their ailments, suffer from not having the legitimacy of the ‘sick role’ [51]. Irrespective of the real illness experiences behind the documents – which we cannot know anything about – we find that some narrators take a chaos perspective, turning the patient’s story into what we have chosen to call the *doctor’s chaos narrative*. In the doctor’s chaos narrative, the physician – implicitly or clearly – is ‘stuck’ with the patient, with no way out of the endless cycle of treatment that leads neither to improved health, nor even, perhaps, to welfare benefits for the patient.

### Conflation of voices

In our material, we find that the voices in the texts are presented in different ways [42]. The narrator may have ‘completeness of information’ about the patient, being an impersonal, authorial, *all-knowing* voice, placed outside the narrative. Then there is *free indirect speech* (no quotation marks or references to the speaker) which may sometimes be inferred as the patient’s voice, given the context of the narrative. Finally, the patient’s voice may be explicit and rendered as *indirect speech* (‘the patient claims/says that…’). The following example is from the certificate of a 50-year-old man with low back pain who has been struggling to convince the NAV official that he is unfit for work

(e) **2.6 Current clinical status (specify date). The results of relevant investigations.** A long talk with the patient […]. For him, it is not possible to resume work because of his low back pain. Still marked by a long fight with NAV for not being believed.

**5.1. Describe how the patient’s functioning is generally reduced due to disease.** Cannot sit or stand for long or short periods of time.

**5.4. What is it that the patient cannot do in the present work?** Cannot function in work, unable to withstand either physical work or sedentary work*.*

In excerpt (e), there are no speaking subjects or references and thus the utterances have no explicit source. The statements in sections 5.1. and 5.4 about the patient’s functionality and working capacity – are they based on the author’s medical observations and knowledge, ‘objectivised’ by the authorial voice to justify disability benefit, or are they the patient’s subjective claims repeated by the narrator in free indirect speech? The conflation of voices erases the distinction between the professional evaluation and the patient’s claims. By using free indirect speech, the narrator has the possibility of not committing to the utterance (which may be inferred as the patient’s own voice) while at the same time making it appear ‘objective’ (inferred as the narrator’s all-knowing voice). The effect in this case is that the narrator can be taken to implicitly promote disability benefits, but without having said it directly.

Only patients can account for their symptoms, and we find that symptoms occupy more space than the diagnosis, which only the doctor is authorised to qualify. Adapting the term *linguistic zone* used by the literary critic Mikhail Bakhtin [52], we consider *symptoms* to belong to the patient’s linguistic universe, whereas *diagnosis* and medical evaluations belong to the doctor’s. In our material, the number of symptoms per certificate ranges from two to fifteen, whereas the number of diagnoses is significantly less, ranging from one to five. By over-emphasising symptoms and complaints, the narrator allows the claimant’s linguistic zone to spread in the text. We find that the corresponding physician’s ‘zone’ within the texts can be displaced or weakened by the narrator’s willingness to leave space for the patient’s linguistic zone, which may perhaps lead the narrator to lose control of the discourse [40].

### The countering voice

Sick leave certificates are often issued by GPs in response to patients’ demands [21, 22, 53]. Some studies indicate that conflict or fear of conflict may underlie the decision to issue a certificate [12, 16]. Certifying permanent working disability probably involves many of the same problems, but few studies have looked at how texts made by doctors when evaluating fitness to work reflect uncertainty and possible conflict with the patient. The writer may use linguistic techniques to point to certain connections or to obscure their own opinion. The example below is from a patient applying for permanent disability benefits after years of receiving temporary benefits. The narrator summarises the patient’s functioning and presents his status in a new certificate below (Certificate (f).We have *italicised* the connecting words):

(f) **Diagnosis:** Epicondylitis L93.

**2.5 History of symptoms and treatment.** Has been doing well in daily life, *but* not managed to resume work. He has not been working *but* has tried to occupy himself with small jobs at home. Painkillers taken occasionally — claims to have some effect — *but* still not ‘healthy’ enough to resume work and wants a prolongation of his disability pension. **8. Optional information**. ‘He has less pain at rest *but* still has pain related to physical efforts.

With the exception of ‘claims to have’ in excerpt (f), which is the patient’s indirect speech, the text contains *free* indirect speech; this speech is probably the patient’s, as it refers to events within the private sphere to which the narrator has no direct access and to the patient’s subjective experience of pain. ‘But’ is a concessive conjunction, which is typical of an argumentative text type; in excerpt (f) it is used systematically to counter statements [54]. We find segments of two opposing narratives, both of which stem from the patient: one referring to his private life (with some achievements) and the other repeatedly to ‘work’ (no achievements). It is not evident why the patient is still unable to work, and the organisation of the text with ‘but’ suggests doubts or disbelief about the patient’s unfitness for work.

Bakhtin [55] points out that the essential quality of an utterance is its *addressiviness*: it is always directed at somebody. In the case of the medical certificates, not only is there the formal receiver of the document, but there are also *super-addressees* – potential readers who are not directly addressed but whom the writer nevertheless has in mind [55]. In excerpt (f), the writer seems to have the patient in mind as a super-addressee; he or she seems to question the patient’s eligibility, but only indirectly, using free indirect style. Using free indirect style here has the double effect of seeming close to the patient while at the same time creating a professional distance [40]. Moreover, putting ‘healthy’ in quotation marks creates a latent irony that must be inferred by the reader, but which may weaken the claimant’s case. Contrary to the narrator in excerpt (e), the narrator in excerpt (f) seems to have doubts about the patient’s claims; none of the narrators, however, expresses their position directly.

### Clarity and ambiguity

Clinical reports usually describe rather than explain. They address readers who have the same professional knowledge as the writer and who are able to infer connections, even in a brief and fragmentary text. In a medical certificate, however, explicit explanation is needed to make the relations between statements clear, as these relations may justify granting benefits. Connective words create meaningful relations between clauses: causal (because, so), adverse (but), temporal (then), or additive (and) words indicate that the text type is explicative, argumentative, narrative, or descriptive, respectively [54, 56]. A document may present with all text types depending on whether the writer’s intention is to explain phenomena, describe facts, tell a story or evaluate/argue. A text may have formal markers showing connections between events, or it may have no markers and still clearly display such connections, as shown in the text below:

(g) **2.5. History of disease, symptoms, and treatment.** She was admitted to hospital. She had back pain, had several falls and paresis. CT showed spinal stenosis*.*

Even though this small text has no markers, we interpret it as recounting a series of events, a narrative. It is also implicitly *causative*: the patient was brought to hospital *because of* her symptoms which were *due to* spinal stenosis. What makes us see this text as coherent, and not as just disconnected clauses, is the co-occurrence of items (back pain – falls – paresis – spinal stenosis), which is a general tendency in texts, known as *collocation.* Accordingly, a competent lay reader will most probably grasp the causal meaning of this text even in the absence of causal conjunctions. In contrast, the additive text type in excerpt (h), below, is similar to that of (g), but unlike (g), (h) creates spurious connections of causality:

(h) **2.5. Illness story, symptoms, treatment.** Pain in the neck, numbness of hands, especially left arm and hand. MRI of the cervical spine shows a minor prolapse at the spinal level C3-C4. **2.6: Current clinical status (specify date). The results of relevant investigations:** The neck mobility is good; no objective, neurologic functional impairments of the arms and hands.

Contrary to excerpt (g), excerpt (h) uses association in a way that can be considered fraudulent. The immediate position of the sentences in relation to one another suggests that the numbness of the left hand is caused by the prolapse at the C3-C4 level of the neck as shown by an MRI. An authorised reader will know that a prolapse at the C3-C4 level of the cervical spine cannot cause general numbness of the hand and therefore will not be surprised that the personal examination of the patient is negative. However, the narrative technique used here, which draws on the reader’s associative ability, is exposed: the text implicitly *suggests* connections that are faulty, even though they seem ‘reasonable on the surface’ [40]. The writer, however, can only be blamed for creating ambiguity, not for deliberately giving faulty information.

### Assessing functionality and working capacity

A lack of information necessary to understand chains of reasoning may create the appearance of incoherence, or even of bias, for the reader. This can create problems in interpretation, especially when there is a request for causal relations between the elements. In excerpt (i), below, the writer neither describes their personal examination of the patient nor provides any other medical results of investigations:

(i) **Diagnosis:** Spondylolisthesis L4. GERD (gastro-oesophageal-reflux-syndrome) D84.

**2.6 Current clinical status (specify date). The results of relevant investigations**. Physical, medical problems causing pain. The patient cannot work. Working is out of the question for the patient. Permanent disease/syndrome with daily symptoms.

In this certificate, detailed medical descriptions explaining how or to what degree the spinal defect and reflux restrict the patient’s bodily and practical daily functions, and how these conditions might explain his reduced working capacity, are replaced by generalisations. In some cases, as in (i), the disease-related diagnosis seems insufficient to explain a total and absolute loss of working ability and appears to serve instead as a Trojan horse of clinical ‘dignity’, or as a legitimate basis of disability benefits. GPs have reported having difficulty describing the impact of subjective health problems on functioning and work ability; they prefer selecting what is most concrete, organ-related, and objectively verifiable [28]. In (i), as in other certificates in our sample, there are various organ-related diagnoses, coded with 70–99 in the ICPC-2 system (International Classification of Primary Care, 2nd edition); however, none of them are accompanied by evaluations of patient functioning that might explain why the patient is unable to work. This might be because no matter the diagnosis, which itself has little to do with the patient’s functional abilities, it is difficult for the doctor to assess the patient’s functioning.

### Breaking of the norm

The fact that ‘disease’ is a non-specific and relative concept, and that NAV might also consider illness as a legitimate basis for benefits – even in the absence of biomedical indicators – leaves a fair amount of room for negotiating eligibility for welfare benefits [4]. Moreover, NAV policy gives more weight to an individual’s degree of impaired functioning than to the medical diagnosis itself [57]. Therefore, deliberately advocating disability pension without any indications of verified disease, illness, or reduced function threatens the narrator’s reliability all the more. In excerpt (j), below, the writer explicitly disproves reduced functioning, yet still suggests that benefits should be considered:

(j) **Diagnosis:** Myalgia L18.

**2.6. Current clinical status (specify date). The results of relevant investigations**. One has, for more than ten years, not managed to get the patient back to work, and therefore, disability pension should be considered. **5.0 Describe how the functioning is generally reduced because of disease**. The patient claims to have myalgia, but there are no somatically reduced functional abilities.

Here, instead of providing the information requested in Section 2.6, the writer immediately introduces the realities of a failed rehabilitation. In Section 5.0, the writer denies that the patient’s functioning is impaired at all. Proposing disability benefits for someone who is not really eligible puts the narrator in conflict with the basic norms of the text – and hence with the implied author (the GP), whose expert standard is expected to comply with the norms displayed in the form text. The writer’s pragmatic suggestion may signify a professional resignation to a long process in which the medical problem is perhaps only of minor importance.

## Discussion

In this paper, we have carried out a close, critical reading of a selection of medical certificates issued in Norway. Whereas other studies show that GPs experience certifying as challenging [8, 17], we have aimed at showing how GPs actually select and mediate information about their patients’ health, functioning, and work capacity. Our text analysis indicates that GPs struggle to provide verifiable, factual information and consistent evaluations. This difficulty manifests in the texts as deficient or lacking information, incoherence (factual and evaluative), absence of professional evaluation, and ambiguity. The GPs’ problems are particularly apparent in their assessments of patient functioning and working capacity regardless of diagnosis, being it subjective illness or ‘organ-related’ disease. The certificates display little insight into working life [8]; however, they rarely express professional uncertainty about patients’ working capacity. Rather, they resort to making general statements and do not report thorough assessments of functioning. Some proved unable to structure and delimit the patient’s illness story, missing the essential causes of the patient’s inability to work. In most cases in our material, it was not clear to us how a particular illness had caused complete and general working disability.

Certifying GPs find the standard form problematic and partly unfit for describing the common sicknesses of our time [28, 58]. We suggest that the form is, to some extent, outdated in relation to the sicknesses and patient-oriented policy of our time and can thus be misleading as to what can in fact be verified by a physician as a cause of working disability. Certifiers seem to struggle to adapt to the form or, more precisely, to the form’s implicit *assumptions* that any illness can be narrated chronologically and that ‘treatment’ is always needed to improve working ability. Subjective illness and chronic pain that cannot be explained by recognised biomedical criteria, and for which there is no effective treatment, create an extra burden for both the patient [51] and the treating GP acting as a certifier [28]. This sometimes results in what we have called the *doctor’s chaos narrative*, reflecting the doctor’s impotence when ‘stuck’ with the patient in a never-ending search for objective findings. In addition, our analysis identifies elements of textual ‘chaos’ throughout the sample texts: disconnected lists of symptoms, missing time aspects, ambiguities and conflation of voices indicating a lack of professional clarity.

The medical certificate is a particular genre of text, hovering on the border of law and medicine. Even though being a certifier engages the GP as part of ‘an apparatus of social justice’ [7], they show little consciousness of the ethics of the medical certificate as a juridical document. According to our analysis, GPs’ ‘strategic writing’ [20] probably entails a variety of chosen linguistic techniques, some of which are subtle. GPs might use subtle writing in the interest of patient advocacy; however, they might also use it to express disagreement with their patients [12]. When it comes to avoiding vagueness and ambiguities, this is not merely a question of textual skills. Rather, we think that such textual failures mirror physicians’ real problems with how to assess, verify and report patients’ functioning and working ability. In addition, there are no specific criteria for assessing impaired functionality – guidelines are few and not mandatory and the medical information can be vague or deficient [59]. Finally, writing a deficient, inaccurate or biased certificate usually carries no negative consequences for the GP.

The mastery of textual skills in a professional genre is not self-evident and should be taught specifically. Physicians in Norway receive no systematic instruction in either medical school or subsequent training on how to produce high quality text in medical certificates. It is probable that medical certificates depend as much on the interest, writing skills, and capacity of the individual certifier as on his/her attitudes and personal values [23]. We suggest that learning in how to write medical certificates (and medical texts in general, which can all be used as juridical documentation) should be integrated as part of the professional curriculum taught in in medical schools. We assume that high quality medical certification strengthens legal protections for the claimant/the patient and reduces arbitrariness in the assessment of disability. Furthermore, we have reason to believe that providing medical certificates with sufficient and necessary information that are accurate, verifiable, and coherent is also economical. Such certificates may facilitate NAV’s work with disability benefit applications, reduce the use of expensive, extra resources and shorten the processing time for disability benefits cases. In addition, the expert’s impartiality and explicitness about his/her uncertainty and limited insight will probably reinforce the legitimacy and reliability of the document. Although a range of other measures is necessary to ensure that benefits are allocated fairly, we believe that GPs taking their role as certifying experts seriously according to the criteria mentioned here will contribute to a fairer evaluation of whether claimants are in fact eligible or not for disability benefits.

On the basis of our findings, it is reasonable to revise the role of GPs as certifiers of fitness for work in Norway. We suggest two possible measures: first, the GPs’ role should be shared with other actors involved in evaluating patients’ treatment and functioning, and the questions posed to the GPs from the NAV should be revised to fit better with the causes of reduced work ability. Second, physicians, medical students, and other health providers involved in certifying health problems should undergo specific instruction in how to write certificates, preferably taught by humanities professors. These measures may improve the quality of certifying documents, and thereby contribute to increasing efficiency and justice in the distribution of public goods.

## Conclusions

Certifying GPs frequently failed to provide clear, sufficient and relevant factual information and coherent medical evaluations to justify the patient’s claims of disability pension. The certificates may not function as reliable sources for making decisions regarding social benefits.

Whereas a deficient certificate has no negative consequences for the certifying GP, it may complicate NAV’s treatment of the case, delay the decision and increase costs by the use of (usually unnecessary) specialists. In addition, insufficient, unclear and biased documentation may lead to wrong or unfair decisions.

Physicians in Norway receive no systematic training in professional writing. We believe that professional writing skills should be validated as an important part of medical practice and should be integrated in medical schools and in further education as a discipline in its own right, preferably involving humanities professors.

### Remarks on method

Compared to our primary selection, we obtained consent to participate from more persons with musculoskeletal diagnosis and from fewer persons with psychiatric diagnosis. However, all main diagnostic categories are present in our material, and we do not think the imbalance in the diagnoses is of importance for the results.

## Abbreviations

GP: General practitioner
NAV: Norwegian Administration of Labour and Welfare
